# Supplementary material for: Prevalence of unqualified sources of antimalarial drug prescription for children under the age of five: A study in 19 low- and middle-income countries
Source: PLoS One. 2024 Mar 21;19(3):e0300347. doi: 10.1371/journal.pone.0300347 (PMC10956821; doi:10.1371/journal.pone.0300347)
Supplement: S1 File — (DOCX) [file pone.0300347.s002.docx]

**Supplementary Materials**

**Methods**

**Statistical Analysis**

We conducted univariate analysis in pooled data to find a significant relationship between the variables of interest and our outcome variable. The existence of a significant relationship between antimalarial prescriptions from qualified sources with selected explanatory variables was examined using univariate logistic regression model. Furthermore, we used binary logistic regression to the pooled data to assess the influence of explanatory variables of antimalarial given to children under five with fever/cough from unqualified sources. The variables identified in the univariate analysis (p-value < 0.20) were then included in the binary logistic regression model.

**Univariate Binary Logistic Regression Models**

**Supplementary table 2** shows the univariate relationship between selected explanatory variables and antimalarial exposures for malaria in children under five. We used proper sampling weight to ensure accurate standard error and p-value estimate in the univariate analysis. We found five explanatory variables in our analysis that are statistically significant at 5% level of significance. The significant explanatory variables are the child’s age, type of place of residence, Number of children under 5 in the household, highest educational level of mother’s and wealth index. The rest of the variables are found to be insignificant.

**Final Binary Logistic Regression Models Adjusted for Country Variations**

**Supplementary table 3** shows the relationship between selected explanatory variables and antimalarial taken from qualified sources for recent fever in children under five. We used proper sampling weight to ensure accurate standard error and p-value estimate in the univariate analysis. In the final binary logistic regression model, we include the variables found to be significant in the univariate analysis (p-value <0.05).

We found that the odds of antimalarial taken from qualified sources for recent fever in children under five were more less likely for the children aged for two years (OR=0.7, 95% CI: 0.6 – 1.0), three years (OR = 0.8, 95% CI: 0.6 –1.1), four years (OR = 0.6, 95% CI: 0.5 – 0.8), and five (OR = 0.6, 95% CI: 0.4 – 0.8) compared to one year. Children whose mothers had primary (OR=0.8, 95% CI:0.6-1.0), secondary (OR=0.7, 95% CI: 0.5-1.0) were less likely but higher education (OR=1.4, 95% CI:0.8-2.5) were more likely to receive antimalarial from qualified sources for recent fever in children under five than those with illiterate mothers. Children whose wealth index had poorer (OR=0.7, 95% CI:0.5-0.9), middle (OR=0.6, 95% CI:0.5-0.8), richer (OR=0.6, 95% CI:0.4-0.8), and richest (OR=0.6, 95% CI:0.4-0.9) were less likely to antimalarial from qualified sources for recent fever in children under five than those with poorest wealth indexed.

**Region Wise Descriptives of Qualified Prescription for Rest of the 9 LMICs**

In Burundi, the overall percentages of antimalarial prescription from qualified sources in children under five for recent malarial fever was high in all regions, with the highest percentages observed in Bujumbur (100%) regions, while the lowest percentages were observed in Ouest (77.9%) regions **[Supplementary figure 1]**. However, the prevalence of antimalarial drug consumption were highest in Centre-East and Ouest regions which was 100% and lowest in Bujumbura Mairie (95.9%) region.

In Guinea, the overall percentages of antimalarial prescription from qualified sources in children under five for recent malarial fever was high in all regions, with the highest percentages observed in Boke (96.3%) region, while the lowest percentages were observed in Kankan (86.4%) region **[Supplementary figure 2]**. However, the prevalence of antimalarial drug consumption was highest in Nzerekore (55.5%) region and lowest in Labe (15.1%) region **[Supplementary table 8]**.

In Madagascar, the overall percentages of antimalarial prescription from qualified sources in children under five for recent malarial fever was high in all regions.The highest percentages observed in Analaman, Atsimo-Andrefana, Atsimo Atsinanana Vakinank, Itasy, Vatovavy, Atsinana, Alaotra, Sofia, Betsibok, Melaky, Anosy, and Sava regions which was about 100% and the lowest percentages were observed in Boeny, Analanji, and Haute Matsiatra (0%) regions [**Supplementary figure 3]**. However, the prevalence of antimalarial drug consumption was highest in Vakinankaratra, Itasy, Haute Matsiatra, Atsimo Atsinanana which is about 100% regions and lowest in Diana 0% region **[Supplementary table 11]**.

In Malawi, the overall percentages of antimalarial prescription from qualified sources in children under five for recent malarial fever was high in all regions. The highest percentages observed in the North (100%) region and the lowest percentage were observed in Central (91.0%) region **[Supplementary figure 4]**. However, the prevalence of antimalarial drug consumption was highest in North, Central which is about 100% regions and lowest in South (99.5%) region **[Supplementary table 12]**.

In Mali, the overall percentages of antimalarial prescription from qualified sources in children under five for recent malarial fever was high in all regions, with the highest percentages observed in Tombouct (100%) region and the lowest percentages were observed in Bamako (74.5%) region **[Supplementary figure 5]**. However, the prevalence of antimalarial drug consumption was highest in Gao (43.6%) region and lowest in kidal (9.6%) region **[Supplementary table 13]**.

In Rwanda, the overall percentages of antimalarial prescription from qualified sources in children under five for recent malarial fever was high in all regions, with the highest percentages observed in West and North regions which about 100% and the lowest percentages were observed in East (87.4%) region [**Supplementary figure 6]**. However, the prevalence of antimalarial drug consumption was 100% in all regions **[Supplementary table 16]**.

In Togo, the overall percentages of antimalarial prescription from qualified sources in children under five for recent malarial fever was high in all regions, with the highest percentages was observed in Aglomeration De Lome, and Kara regions which was 100% and the lowest Maritime (87.9%) **[Supplementary figure 7]**. However, the prevalence of antimalarial drug consumption was 100% except for Maritime (95.7%), and Centrale (90.9%), regions.

In Uganda, the overall percentages of antimalarial prescription from qualified sources in children under five for recent malarial fever was high in all regions, with the highest percentages observed in Karamoja (100%) region, while the lowest percentages were observed in Kampala (87.9%) regions **[Supplementary figure 8]**. However, the prevalence of antimalarial drug consumption was 100% except for North Buganda (94.4%), Teso(97.4%), Acholi (99.4%), West Nile (98.8%), regions **[Supplementary table 21]**.

In Zambia, the overall percentages of antimalarial prescription from qualified sources in children under five for recent malarial fever was high in all regions, with the highest percentages observed in Copperbelt (100%) region, while the lowest percentages were observed in Southern (73.3%) region [**Supplementary figure 9]**. However, the prevalence of antimalarial drug consumption was 100% except for Central (97.3%), and Luapula (98.4%) regions **[Supplementary table 22]**.

**Supplementary tables 1-22**

**Supplementary tables 1:** Variables Recoding Procedures of MIS Datasets

| **Variables** | **Code at MIS Dataset** | **Categories in MIS** | **Recoding Procedure** |
| --- | --- | --- | --- |
| **Level One Variables (Individual Level Variables)** | | | |
| Number of children under 5 | v137 | One | Recoded as 1= "one", 2 ="two", 3= "three ", 4= "four", 5= "five or above". |
|  |  | Two |  |
|  |  | Three |  |
|  |  | Four |  |
|  |  | Five or above |  |
| Age of the child (In Years) | hw1 | One year old | Recoded as 1 = “min/12months”, 2= “13/24months”, 3 = “25/36months”, 4= “37/48months”, and 5 =”49/60months”. |
|  |  | Two years old |  |
|  |  | Three years old |  |
|  |  | Four years old |  |
|  |  | Five years old |  |
| Sex of the child | b4 | Male | Recoded as 1 =” male” and 2= “female” |
|  |  | female |  |
| Wealth index | v190 | Poorest | Recoded as 1= “poorest”, 2= “poorer”, 3 = “middle”, 4= “richer” and 5= “richest” |
|  |  | Poorer |  |
|  |  | Middle |  |
|  |  | Richer |  |
|  |  | Richest |  |
| Had fever in last two weeks | h22 | Yes | Recoded as  1 = “Yes”  0 = “No” |
|  |  | No |  |
| Taken antimalarial drugs for recent malarial fever | this was deducted by code from the dataset. | Yes | Recoded as  1 = “Yes”  0 = “No” |
|  |  | No |  |
| Mother’s highest level of education | v106 | No education | Recoded as 0= “no education”, 1= “primary”, 2 = “secondary”, and 3= “Higher” |
|  |  | Primary |  |
|  |  | Secondary |  |
|  |  | Higher |  |
| **Level Two Variables (Community Variables)** | | | |
| Type of place of Residence | v025 | Urban | Recoded as  1 = “Urban”  2 = “Rural” |
|  |  | Rural |  |
| Country Code | v000 | This cell needs a hand |  |
| **Dependent Variables** | | | |
| Antimalarial drugs taken from qualified sources for recent malarial fever. | this was deducted by code from the dataset. | Yes | Recoded as  1 = “Yes”  0 = “No” |
|  |  | No |  |
| **Random Effect Variables** | | | |
| Sampling Weight | v005 | For weighting the observation |  |
| Primary sampling unit (PSU) | v021 | For sample selection |  |
| Strata | v022 | For stratification |  |

**Supplementary table 2:** Univariate Analysis of Socio-economic Variables Associated with Qualified Prescriptions of Antimalarial for recent malarial fever in 19 LMICs.

| **Variables** | **Category** | **OR** | **P-Value** | **CI** |
| --- | --- | --- | --- | --- |
| Child’s age | One year old | **Ref.** | **Ref.** | **Ref.** |
|  | Two years old | 0.8 | 0.17 | [0.6-1.1] |
|  | Three years old | 0.9 | 0.26 | [0.7-1.1] |
|  | Four years old | 0.7 | <0.01 | [0.5-0.8] |
|  | Five years old | 0.7 | <0.01 | [0.5-0.9] |
| Sex of the Child | Male | **Ref.** | **Ref.** | **Ref.** |
|  | Female | 1.0 | 0.67 | [0.9-1.2] |
| Type of place of residence | Urban | **Ref.** | **Ref.** | **Ref.** |
|  | Rural | 1.4 | <0.01 | [1.1-1.8] |
| Highest educational level of Mother’s | No education | **Ref.** | **Ref.** | **Ref.** |
|  | Primary | 0.7 | <0.01 | [0.6-0.8] |
|  | Secondary | 0.4 | <0.01 | [0.3-0.5] |
|  | Higher | 0.5 | <0.01 | [0.3-0.7] |
| Number of children under 5 in the household | One | **Ref.** | **Ref.** | **Ref.** |
|  | Two | 1.3 | <0.01 | [1.1-1.6] |
|  | Three | 0.9 | 0.54 | [0.7-1.2] |
|  | Four | 0.9 | 0.77 | [0.5-1.6] |
|  | Five or above | 1.2 | 0.32 | [0.8-1.9] |
| Wealth index combined | Poorest | **Ref.** | **Ref.** | **Ref.** |
|  | Poorer | 0.7 | <0.01 | [0.5-0.9] |
|  | Middle | 0.7 | <0.01 | [0.5-0.9] |
|  | Richer | 0.5 | <0.01 | [0.4-0.7] |
|  | Richest | 0.5 | <0.01 | [0.4-0.7] |

**Supplementary table 3:** Final Binary Logistic Regression Model for Pooled data Adjusted for Country Variations.

| **Variables** | **Category** | **OR** | **P-Value** | **CI** |
| --- | --- | --- | --- | --- |
| Child’s age | One year old | **Ref.** | **Ref.** | **Ref.** |
|  | Two years old | 0.7 | 0.04 | [0.6-1.0] |
|  | Three years old | 0.8 | 0.24 | [0.6-1.1] |
|  | Four years old | 0.6 | <0.01 | [0.5-0.8] |
|  | Five years old | 0.6 | <0.01 | [0.4-0.8] |
| Type of place of residence | Urban | **Ref.** | **Ref.** | **Ref.** |
|  | Rural | 1.0 | 0.92 | [0.8-1.3] |
| Highest educational level of Mother’s | No education | **Ref.** | **Ref.** | **Ref.** |
|  | Primary | 0.8 | 0.11 | [0.6-1.0] |
|  | Secondary | 0.7 | 0.05 | [0.5-1.0] |
|  | Higher | 1.4 | 0.23 | [0.8-2.5] |
| Number of children under 5 in the household | One | **Ref.** | **Ref.** | **Ref.** |
|  | Two | 1.2 | 0.12 | [1.0-1.5] |
|  | Three | 0.9 | 0.42 | [0.7-1.2] |
|  | Four | 0.9 | 0.59 | [0.5-1.4] |
|  | Five or above | 1.0 | 0.92 | [0.6-1.7] |
| Wealth index  combined | Poorest | **Ref.** | **Ref.** | **Ref.** |
|  | Poorer | 0.7 | 0.01 | [0.5-0.9] |
|  | Middle | 0.6 | <0.01 | [0.5-0.8] |
|  | Richer | 0.6 | <0.01 | [0.4-0.8] |
|  | Richest | 0.6 | 0.01 | [0.4-0.9] |

**Region-wise Antimalarial Qualified Percentage for 19 Countries**

**Supplementary table 4:** Weighted Descriptive Statistics for Each Region in Angola

| **Name of the Region** | **Antimalarial Taken for Malarial Fever, N (%)** | **Antimalarial Taken from Qualified Sources, N (%)** | **Antimalarial Taken from Unqualified sources, N (%)** |
| --- | --- | --- | --- |
| Hyperendemic | 86.1 (94.2) | 78.6 (98.8) | 0.9 (1.1) |
| Mesoendemic Stable | 299.1 (96.2) | 280.5 (98.8) | 3.2 (1.1) |
| Mesoendemic Unstable | 111.5 (95.1) | 102.2 (95.5) | 4.8 (4.4) |
| Luanda | 209.4 (93.5) | 194.9 (100) | 0 (0) |

**Supplementary table 5:** Weighted Descriptive Statistics for Each Region in Burkina Faso

| **Name of the Region** | **Antimalarial Taken for Malarial Fever, N (%)** | **Antimalarial Taken from Qualified Sources, N (%)** | **Antimalarial Taken from Unqualified sources, N (%)** |
| --- | --- | --- | --- |
| Boucle du Mouhoun | 37.0 (100) | 34.6 (93.5) | 2.4 (6.5) |
| Cascades | 41.6 (100) | 39.5 (95.9) | 1.6 (4.0) |
| Centre | 93.5 (97.0) | 30.1 (100) | 0 (0) |
| Centre-Est | 97.4 (100) | 82.6 (100) | 0 (0) |
| Centre-Nord | 67.4 (100) | 63.4 (100) | 0 (0) |
| Centre-Ouest | 42.7 (100) | 38.2 (93.1) | 2.8 (6.8) |
| Centre-Sud | 10.6 (100) | 9.6 (94.7) | 0.5 (5.2) |
| Est | 59.3 (100) | 58.5 (100) | 0 (0) |
| Hauts-Bassins | 64.2 (100) | 56.9 (91.6) | 5.2 (8.4) |
| Nord | 55.8 (100) | 54.9 (100) | 0 (0) |
| Plateau Central | 29.1 (97.9) | 28.4 (97.7) | 0.6 (2.3) |
| Sahel | 33.9 (100) | 30.9 (95.9) | 1.3 (4.1) |
| Sud-Ouest | 29.4 (98.1) | 28.9 (100) | 0 (0) |

**Supplementary table 6:** Weighted Descriptive Statistics for Each Region in Burundi

| **Name of the Region** | **Antimalarial Taken for Malarial Fever, N (%)** | **Antimalarial Taken from Qualified Sources, N (%)** | **Antimalarial Taken from Unqualified sources, N (%)** |
| --- | --- | --- | --- |
| Bujumbura Mairie | 10.6 (95.9) | 9.8 (100) | 0 (0) |
| North | 158.2 (96.8) | 145.3 (93.7) | 9.7 (6.3) |
| Central East | 132.8 (100) | 122.3 (96.9) | 3.9 (3.1) |
| West | 66.3 (100) | 49.6 (77.9) | 14.0 (22.0) |
| South | 87.5 (96.7) | 77.2 (91.6) | 7.0 (8.3) |

**Supplementary table 7:** Weighted Descriptive Statistics for Each Region in Ghana

| **Name of the Region** | **Antimalarial Taken for Malarial Fever, N (%)** | **Antimalarial Taken from Qualified Sources, N (%)** | **Antimalarial Taken from Unqualified sources, N (%)** |
| --- | --- | --- | --- |
| Upper East | 22.9 (100) | 19.0 (96.6) | 0.6 (3.4) |
| Western | 74.4 (98.8) | 37.5 (63.0) | 22.0 (36.9) |
| Upper West | 10.8 (100) | 8.2 (82.4) | 1.7 (17.5) |
| Northern | 66.5 (100) | 44.5 (82.4) | 9.4 (17.5) |
| Brong-Ahafo | 32.7 (100) | 21.6 (79.8) | 5.4 (20.1) |
| Volta | 31.8 (100) | 14.6 (51.0) | 14.1 (48.9) |
| Central | 29.1 (100) | 15.3 (59.9) | 10.2 (40.0) |
| Greater Accra | 27.1 (100) | 6.2 (23.1) | 20.8 (76.8) |
| Ashanti | 39.3 (100) | 12.3 (33.5) | 24.5 (66.5) |
| Eastern | 35.3 (100) | 18.3 (51.7) | 17.0 (48.2) |

**Supplementary table 8:** Weighted Descriptive Statistics for Each Region in Guinea

| **Name of the Region** | **Antimalarial Taken for Malarial Fever, N (%)** | **Antimalarial Taken from Qualified Sources, N (%)** | **Antimalarial Taken from Unqualified sources, N (%)** |
| --- | --- | --- | --- |
| Boké | 45.0 (41.6) | 37.9 (96.3) | 1.4 (3.6) |
| Conakry | 21.4 (18.1) | 16.3 (90.5) | 1.7 (9.4) |
| Faranah | 33.3 (42.6) | 27.7 (93.2) | 2.0 (6.7) |
| Kankan | 52.3 (26.4) | 42.5 (86.3) | 6.7 (13.6) |
| Kindia | 32.3 (23.2) | 24.1 (92.0) | 2.1 (8.0) |
| Labé | 13.8 (15.1) | 10.4 (87.5) | 1.5 (12.4) |
| Mamou | 19.3 (42.0) | 13.7 (87.5) | 1.9 (12.5) |
| N'Zérékoré | 72.0 (55.5) | 62.9 (93.3) | 4.5 (6.6) |

**Supplementary table 9:** Weighted Descriptive Statistics for Each Region in Kenya

| **Name of the Region** | **Antimalarial Taken for Malarial Fever, N (%)** | **Antimalarial Taken from Qualified Sources, N (%)** | **Antimalarial Taken from Unqualified sources, N (%)** |
| --- | --- | --- | --- |
| Coast | 2.2 (3.1) | 1.3 (59.4) | 0.9 (40.6) |
| North Eastern | 0 (0) | - | - |
| Eastern | 0.6 (2.3) | 0.4 (100) | 0 (0) |
| Central | 0 (0) | - | - |
| Rift Valley | 5.7 (4.6) | 4.2 (75.1) | 1.4 (24.9) |
| Western | 2.5 (2.5) | 2.1 (100) | 0 (0) |
| Nyanza | 3.7 (3.1) | 3.4 (100) | 0 (0) |
| Nairobi | 0 (0) | - | - |

**Supplementary table 10:** Weighted Descriptive Statistics for Each Region in Liberia

| **Name of the Region** | **Antimalarial Taken for Malarial Fever, N (%)** | **Antimalarial Taken from Qualified Sources, N (%)** | **Antimalarial Taken from Unqualified sources, N (%)** |
| --- | --- | --- | --- |
| Greater Monrovia | 167.0 (95.1) | 99.4 (61.1) | 63.3 (38.9) |
| North Western | 74.7 (96.0) | 63.9 (90.4) | 6.8 (9.6) |
| South Central | 101.3 (94.7) | 64.4 (72.2) | 24.7 (27.7) |
| South Eastern A | 38.8 (100) | 30.9 (87.7) | 4.3 (12.2) |
| South Eastern B | 37.3 (96.9) | 32.0 (93.0) | 2.4 (7.0) |
| North Central | 223.1 (99.2) | 155.7 (76.6) | 47.5 (23.3) |

**Supplementary table 11:** Weighted Descriptive Statistics for Each Region in Madagascar

| **Name of the Region** | **Antimalarial Taken for Malarial Fever, N (%)** | **Antimalarial Taken from Qualified Sources, N (%)** | **Antimalarial Taken from Unqualified sources, N (%)** |
| --- | --- | --- | --- |
| Analamanga excluding capital | 6.2 (48.2) | 6.3 (100) | 0 (0) |
| Vakinankaratra | 1.1 (100) | 0.7 (100) | 0 (0) |
| Itasy | 1.3 (100) | 1.3 (100) | 0 (0) |
| Bongolava | - | - | - |
| Haute Matsiatra | 3.2 (100) | 0 (0) | 3.2 (100) |
| Amoron'i Mania | 1.1 (48.8) | () | () |
| Vatovavy Fitovinany | 8.0 (65.7) | 6.1 (100) | 0 (0) |
| Ihorombe | - | - | - |
| Atsimo Atsinanana | 4.4 (100) | 3.8 (100) | 0 (0) |
| Atsinanana | 3.9 (60.9) | 3.9 (100) | 0 (0) |
| Analanjirofo | 1.8 (67.5) | 0 (0) | 1.3 (100) |
| Alaotra Mangoro | 2.5 (28.9) | 2.5 (100) | 0 (0) |
| Boeny | 1.5 (29.2) | 0 (0) | 1.5 (100) |
| Sofia | 6.6 (60.4) | 5.3 (100) | 0 (0) |
| Betsiboka | 1.3 (47.6) | 1.3 (100) | 0 (0) |
| Melaky | 2.5 (73.1) | 1.0 (100) | 0 (0) |
| Atsimo Andrefana | 2.3 (32.8) | 2.1 (100) | 0 (0) |
| Androy | 2.5 (74.8) | 2.3 (91.6) | 0.2 (8.4) |
| Anosy | 11.1 (92.8) | 11.1 (100) | 0 (0) |
| Menabe | 3.5 (66.4) | 2.2 (71.8) | 0.8 (28.2) |
| Diana | 0 (0) | - | - |
| Sava | 2.9 (68.1) | 1.3 (100) | 0 (0) |
| Antananarivo capital | - | - | - |

**Supplementary table 12:** Weighted Descriptive Statistics for Each Region in Malawi

| **Name of the Region** | **Antimalarial Taken for Malarial Fever, N (%)** | **Antimalarial Taken from Qualified Sources, N (%)** | **Antimalarial Taken from Unqualified sources, N (%)** |
| --- | --- | --- | --- |
| North | 16.4 (100) | 14.1 (100) | 0 (0) |
| Central | 170.7 (100) | 147.3 (91.0) | 14.5 (8.9) |
| South | 118.3 (99.5) | 86.3 (93.6) | 5.9 (6.4) |

**Supplementary table 13:** Weighted Descriptive Statistics for Each Region in Mali

| **Name of the Region** | **Antimalarial Taken for Malarial Fever, N (%)** | **Antimalarial Taken from Qualified Sources, N (%)** | **Antimalarial Taken from Unqualified sources, N (%)** |
| --- | --- | --- | --- |
| Kayes | 106.6 (21.6) | 101.0 (96.6) | 3.5 (3.3) |
| Koulikoro | 161.6 (42.8) | 154.5 (99.0) | 1.6 (1.0) |
| Sikasso | 149.5 (31.2) | 128.3 (92.3) | 10.6 (7.6) |
| Segou | 163.6 (38.7) | 147.6 (92.9) | 11.2 (7.0) |
| Mopti | 86.3 (26.7) | 70.5 (92.8) | 5.5 (7.2) |
| Tombouctou | 28.8 (31.8) | 28.3 (100) | 0 (0) |
| Gao | 19.8 (43.6) | 12.1 (84.9) | 2.1 (15.1) |
| Kidal | 0.9 (9.5) | 0.1 (80.7) | 0.01 (19.2) |
| Bamako | 63.1 (23.9) | 41.8 (74.4) | 14.3 (25.5) |

**Supplementary table 14:** Weighted Descriptive Statistics for Each Region in Mozambique

| **Name of the Region** | **Antimalarial Taken for Malarial Fever, N (%)** | **Antimalarial Taken from Qualified Sources, N (%)** | **Antimalarial Taken from Unqualified sources, N (%)** |
| --- | --- | --- | --- |
| Niassa | 102.4 (100) | 33.0 (100) | 0 (0) |
| Cabo Delgado | 95.2 (100) | 33.2 (93.8) | 2.1 (6.1) |
| Nampula | 349.1 (100) | 94.7 (95.2) | 4.7 (4.8) |
| Zambezia | 388.4 (100) | 126.8 (100) | 0 (0) |
| Tete | 134.8 (100) | 43.1 (100) | 0 (0) |
| Manica | 76.4 (100) | 27.7 (100) | 0 (0) |
| Sofala | 112.1 (100) | 26.0 (100) | 0 (0) |
| Inhambane | 65.2 (100) | 24.9 (96.0) | 1.0 (3.9) |
| Gaza | 52.7 (100) | 9.8 (100) | 0 (0) |
| Maputo Provincia | 50.3 (100) | 1.6 (100) | 0 (0) |
| Maputo Cidade | 31. 9(100) | 1.3 (100) | 0 (0) |

**Supplementary table 15:** Weighted Descriptive Statistics for Each Region in Nigeria

| **Name of the Region** | **Antimalarial Taken for Malarial Fever, N (%)** | **Antimalarial Taken from Qualified Sources, N (%)** | **Antimalarial Taken from Unqualified sources, N (%)** |
| --- | --- | --- | --- |
| Sokoto | 15.9 (6.0) | 8.2 (58.5) | 5.8 (41.5) |
| Zamfara | 21.0 (21.1) | 9.9 (52.8) | 8.8 (47.1) |
| Katsina | 12.4 (4.2) | 8.1 (65.8) | 4.2 (34.1) |
| Jigawa | 15.6 (6.8) | 14.7 (100) | 0 (0) |
| Yobe | 25.6 (15.8) | 12.7 (68.5) | 5.8 (31.4) |
| Borno - Urban | 1.7 (2.9) | 1.1 (63.4) | 0.6 (36.5) |
| Adamawa | 15.4 (27.2) | 10.7 (78.2) | 2.9 (21.7) |
| Gombe | 4.9 (13.2) | 2.3 (72.4) | 0.9 (27.6) |
| Bauchi | 86.6 (27.6) | 50.8 (69.4) | 22.3 (30.5) |
| Kano | 26.3 (10.6) | 16.2 (73.0) | 6.0 (27.0) |
| Kaduna | 20.3 (7.0) | 14.4 (77.0) | 4.3 (23.0) |
| Kebbi | 1.5 (0.4) | 1.5 (100) | 0 (0) |
| Niger | 35.0 (16.3) | 20.3 (81.6) | 4.6 (18.4) |
| FCT Abuja | 28.0 (54.3) | 19.6 (84.2) | 3.6 (15.7) |
| Nasarawa | 20.6 (46.6) | 10.4 (65.1) | 5.6 (34.9) |
| Plateau | 2.0 (5.5) | 0.4 (100) | 0 (0) |
| Taraba | 17.9 (32.3) | 9.0 (67.7) | 4.3 (32.3) |
| Benue | 35.4 (73.4) | 11.3 (36.0) | 20.0 (63.9) |
| Kogi | 24.0 (55.7) | 15.5 (72.6) | 5.8 (27.3) |
| Kwara | 19.1 (42.9) | 9.5 (59.5) | 6.4 (40.4) |
| Oyo | 25.5 (48.0) | 16.9 (100) | 0 (0) |
| Osun | 10.6 (43.1) | 6.1 (61.8) | 3.7 (38.1) |
| Ekiti | 5.3 (43.5) | 2.2 (59.9) | 1.5 (40.1) |
| Ondo | 4.4 (33.5) | 1.9 (48.1) | 2.0 (51.8) |
| Edo | 15.9 (33.9) | 8.5 (60.3) | 5.6 (39.6) |
| Anambra | 43.2 (70.5) | 14.8 (36.5) | 25.6 (63.4) |
| Enugu | 16.7 (48.8) | 7.8 (52.5) | 7.0 (47.4) |
| Ebonyi | 34.6 (43.5) | 10.3 (32.3) | 21.6 (67.6) |
| Cross River | 27.9 (43.8) | 9.2 (51.2) | 8.7 (48.7) |
| Akwa Ibom | 13.1 (12.2) | 3.6 (35.4) | 6.6 (64.5) |
| Abia | 34.2 (55.2) | 8.5 (29.4) | 20.5 (70.6) |
| Imo | 17.0 (40.5) | 5.8 (41.8) | 8.0 (58.1) |
| Rivers | 18.6 (28.6) | 3.3 (22.6) | 11.5 (77.3) |
| Bayelsa | 3.5 (14.6) | 1.5 (50.2) | 1.5 (49.7) |
| Delta | 29.6 (29.6) | 10.5 (57.3) | 7.8 (42.6) |
| Lagos | 54.7 (45.8) | 30.6 (64.5) | 16.8 (35.5) |
| Ogun | 14.2 (34.0) | 5.4 (81.6) | 1.2 (18.3) |

**Supplementary table 16:** Weighted Descriptive Statistics for Each Region in Rwanda

| **Name of the Region** | **Antimalarial Taken for Malarial Fever, N (%)** | **Antimalarial Taken from Qualified Sources, N (%)** | **Antimalarial Taken from Unqualified sources, N (%)** |
| --- | --- | --- | --- |
| City of Kigali | 0 (100) | 7.9 (88.8) | 1.0 (11.1) |
| South | 0 (100) | 38.7 (96.9) | 1.2 (3.1) |
| West | 0 (100) | 24.4 (100) | 0 (0) |
| North | 0 (100) | 4.7 (100) | 0 (0) |
| East | 0 (100) | 77.4 (87.4) | 11.1 (12.6) |

**Supplementary table 17:** Weighted Descriptive Statistics for Each Region in Senegal

| **Name of the Region** | **Antimalarial Taken for Malarial Fever, N (%)** | **Antimalarial Taken from Qualified Sources, N (%)** | **Antimalarial Taken from Unqualified sources, N (%)** |
| --- | --- | --- | --- |
| Dakar | 0 (0) | 0 (0) | 0(0) |
| Diourbel | 21.1 (6.3) | 21.1 (6.3) | 0(0) |
| Fatick | 0 (0) | 0 (0) | 0(0) |
| Kaffrine | 3.1 (1.5) | 3.1 (1.5) | 0(0) |
| Kaolack | 0 (0) | 0 (0) | 0(0) |
| Kedougou | 0.5 (2.6) | 0.5 (2.6) | 0(0) |
| Kolda | 3.8 (3.9) | 3.8 (3.9) | 0(0) |
| Louga | 0.9 (0.7) | 0.9 (0.7) | 0(0) |
| Matam | 2.4 (7.3) | 2.4 (7.3) | 0(0) |
| Saint-Louis | 2.7 (3.0) | 2.7 (3.0) | 0(0) |
| Sedhiou | 2.3 (9.6) | 2.3 (9.6) | 0(0) |
| Tambacounda | 6.1 (7.2) | 6.1 (7.2) | 0(0) |
| Thies | 0 (0) | 0 (0) | 0(0) |
| Ziguinchor | 0.7 (2.7) | 0.7 (2.7) | 0(0) |

**Supplementary table 18:** Weighted Descriptive Statistics for Each Region Sierra Leone

| **Name of the Region** | **Antimalarial Taken for Malarial Fever, N (%)** | **Antimalarial Taken from Qualified Sources, N (%)** | **Antimalarial Taken from Unqualified sources, N (%)** |
| --- | --- | --- | --- |
| Eastern | 215.2(24.8) | 197.5(98.7) | 2.6(1.3) |
| Northern | 323.0 (37.2) | 289.9(97.4) | 7.8(2.6) |
| Southern | 239.2 (27.6) | 210.6(94.0) | 13.5(6.0) |
| Western | 90.8(10.5) | 65.9(96.0) | 2.7(4.0) |

**Supplementary table 19:** Weighted Descriptive Statistics for Each Region in Tanzania

| **Name of the Region** | **Antimalarial Taken for Malarial Fever, N (%)** | **Antimalarial Taken from Qualified Sources, N (%)** | **Antimalarial Taken from Unqualified sources, N (%)** |
| --- | --- | --- | --- |
| Dodoma | 1.8 (100) | 1.8 (100) | 0 (0) |
| Arusha | 2.4 (100) | 2.4 (100) | 0 (0) |
| Kilimanjaro | - | - | - |
| Tanga | 8.6 (100) | 6.7 (77.3) | 1.9 (22.6) |
| Morogoro | 33.4 (100) | 16.9 (53.2) | 14.9 (46.8) |
| Pwani | 5.7 (96.8) | 3.6 (63.4) | 2.1 (36.5) |
| Dar es Salaam | 15.7 (100) | 15.7 (100) | 0 (0) |
| Lindi | 5.3 (100) | 4.3 (94.3) | 0.2 (5.7) |
| Mtwara | 21.7 (100) | 17.8 (84.8) | 3.1 (15.1) |
| Ruvuma | 15.8 (100) | 6.0 (44.2) | 7.5 (55.8) |
| Iringa | 4.2 (100) | 3.3 (78.3) | 1.0 (21.6) |
| Mbeya | 3.5 (100) | 1.8 (51.4) | 1.7 (48.5) |
| Singida | 15.8 (100) | 4.6 (41.6) | 6.5 (58.4) |
| Tabora | 61.0 (95.6) | 18.9 (42.6) | 25.4 (57.3) |
| Rukwa | 52.0 (100) | 29.0 (55.9) | 22.9 (44.1) |
| Kigoma | 43.5 (100) | 21.5 (61.9) | 13.2 (38.0) |
| Shinyanga | 16.7 (97.1) | 6.9 (46.1) | 8.0 (53.8) |
| Kagera | 19.4 (100) | 10.6 (80.3) | 2.6 (19.6) |
| Mwanza | 51.8 (100) | 33.4 (77.0) | 10.0 (23.0) |
| Mara | 50.9 (100) | 18.3 (47.2) | 20.5 (52.7) |
| Manyara | 3.4 (100) | 1.3 (55.7) | 1.0 (44.3) |
| Njombe | 0.8 (100) | 0.8 (100) | 0 (0) |
| Katavi | 6.7 (100) | 3.4 (57.1) | 2.5 (42.8) |
| Simiyu | 23.1 (100) | 9.4 (44.0) | 12.0 (56.0) |
| Geita | 37.3 (100) | 14.9 (62.0) | 9.1 (37.9) |
| Kaskazini Unguja | - | - | - |
| Kusini Unguja | - | - | - |
| Mjini Magharibi | - | - | - |
| Kaskazini Pemba | - | - | - |
| Kusini Pemba | - | - | - |

**Supplementary table 20:** Weighted Descriptive Statistics for Each Region in Togo

| **Name of the Region** | **Antimalarial Taken for Malarial Fever, N (%)** | **Antimalarial Taken from Qualified Sources, N (%)** | **Antimalarial Taken from Unqualified sources, N (%)** |
| --- | --- | --- | --- |
| Centrale | 15.5 (90.9) | 13.7 (93.0) | 1.0 (7.0) |
| Agglomération de Lomé | 40.2 (100) | 31.1 (100) | 0 (0) |
| Kara | 19.1 (100) | 17.9 (100) | 0 (0) |
| Maritime | 49.4 (95.7) | 40.2 (87.9) | 5.5 (12.1) |
| Plateaux | 46.0 (100) | 37.7 (91.8) | 3.3 (8.1) |
| Savanes | 54.8 (100) | 49.4 (98.2) | 0.8 (1.7) |

**Supplementary table 21:** Weighted Descriptive Statistics for Each Region in Uganda

| **Name of the Region** | **Antimalarial Taken for Malarial Fever, N (%)** | **Antimalarial Taken from Qualified Sources, N (%)** | **Antimalarial Taken from Unqualified sources, N (%)** |
| --- | --- | --- | --- |
| Acholi | 80.6 (99.4) | 69.5 (94.2) | 4.2 (5.7) |
| Ankole | 32.4 (100) | 27.9 (92.5) | 2.2 (7.5) |
| Bugisu | 50.9 (100) | 38.8 (84.0) | 7.3 (15.9) |
| Bukedi | 31.3 (100) | 24.3 (83.0) | 5.0 (17.0) |
| Bunyoro | 48.5 (100) | 31.9 (73.2) | 11.6 (26.7) |
| Busoga | 116.0 (100) | 94.1 (88.1) | 12.6 (11.8) |
| North Buganda | 135.0 (94.4) | 112.9 (89.9) | 12.7 (10.1) |
| South Buganda | 73.8 (100) | 62.4 (87.0) | 9.3 (13.0) |
| Kampala | 7.5 (100) | 4.2 (66.8) | 2.1 (33.2) |
| Karamoja | 27.5 (100) | 27.5 (100) | 0 (0) |
| Kigezi | 15.3 (100) | 13.9 (95.7) | 0.6 (4.2) |
| Lango | 80.3 (100) | 75.7 (96.9) | 2.3 (3.0) |
| Teso | 90.1 (97.3) | 66.0 (83.1) | 13.3 (16.8) |
| Tooro | 119.8 (100) | 83.8 (74.0) | 29.3 (25.9) |
| West Nile | 165.1 (98.8) | 150.0 (94.1) | 9.3 (5.8) |

**Supplementary table 22:** Weighted Descriptive Statistics for Each Region in Zambia

| **Name of the Region** | **Antimalarial Taken for Malarial Fever, N (%)** | **Antimalarial Taken from Qualified Sources, N (%)** | **Antimalarial Taken from Unqualified sources, N (%)** |
| --- | --- | --- | --- |
| Central | 77.1 (97.3) | 61.0 (87.5) | 8.7 (12.5) |
| Copperbelt | 125.4 (100) | 118.7 (100) | 0 (0) |
| Eastern | 158.9 (100) | 128.3 (89.8) | 15.0 (10.1) |
| Luapula | 194.6 (98.3) | 177.2 (96.4) | 6.4 (3.5) |
| Lusaka | 47.2 (100) | 32.1 (85.0) | 5.6 (14.9) |
| Muchinga | 87.2 (100) | 71.7 (95.3) | 3.5 (4.6) |
| North Western | 111.6 (100) | 105.4 (97.8) | 2.3 (2.1) |
| Northern | 211.8 (100) | 181.8 (92.3) | 15.0 (7.6) |
| Southern | 31.5 (100) | 20.2 (73.2) | 7.3 (26.7) |
| Western | 35.2 (100) | 27.8 (88.1) | 3.7 (11.8) |

**Supplementary figures 1-9**

**Supplementary figure 1:** Region-wise Prevalence of Qualified Prescription of Antimalarial from Qualified Sources in Burundi

**
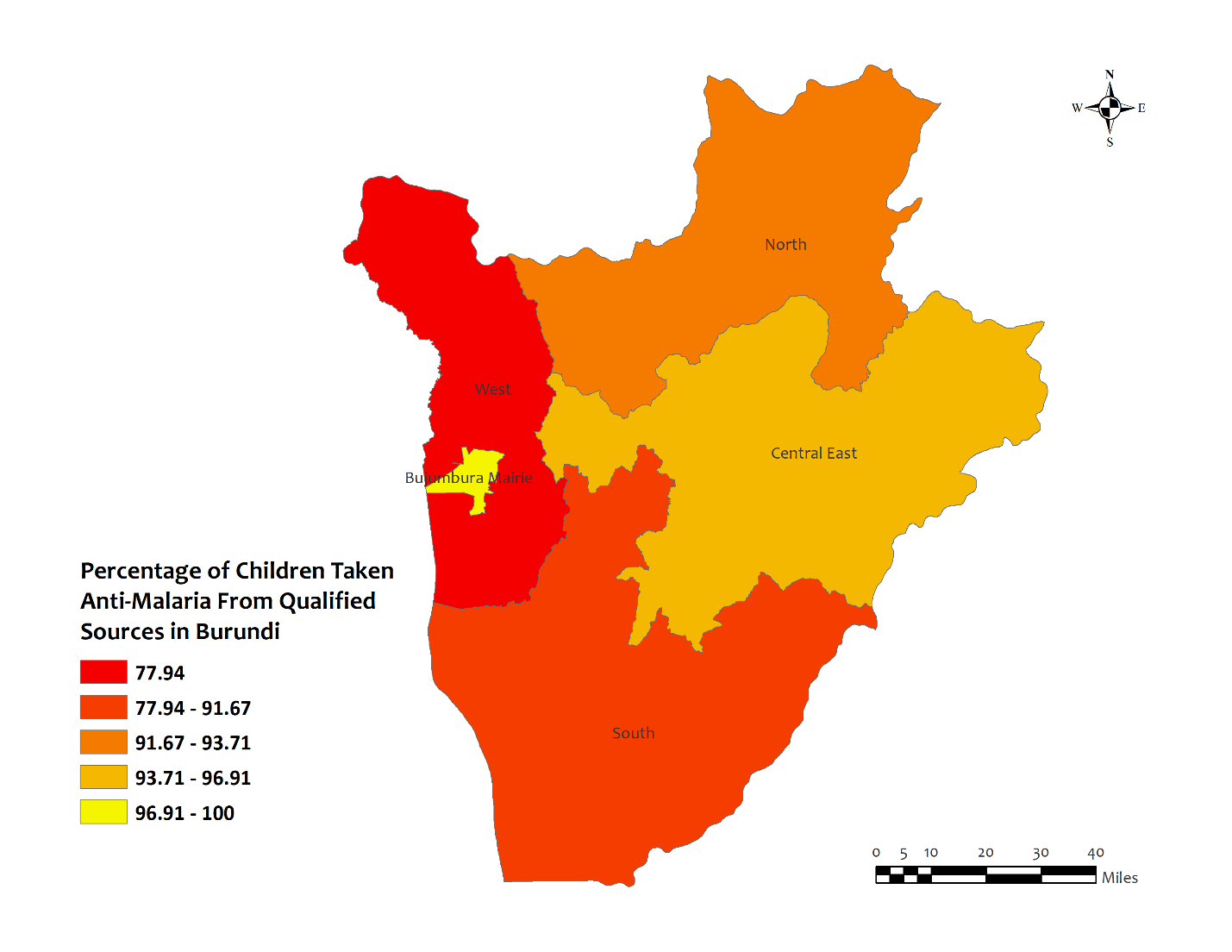
**

***Legend:*** ***Here, the darker shades of red indicate unqualified sources of antimalarial.*** ***Basemap data provided by ArcGIS (source:***[***https://hub.arcgis.com/datasets/esri::world-countries-generalized/explore?location=76.272878%2C-132.879863%2C12.88***](https://hub.arcgis.com/datasets/esri::world-countries-generalized/explore?location=76.272878%2C-132.879863%2C12.88)***)***

**Supplementary figure 2:** Region-wise Prevalence of Qualified Prescription of Antimalarial from Qualified Sources in Guinea


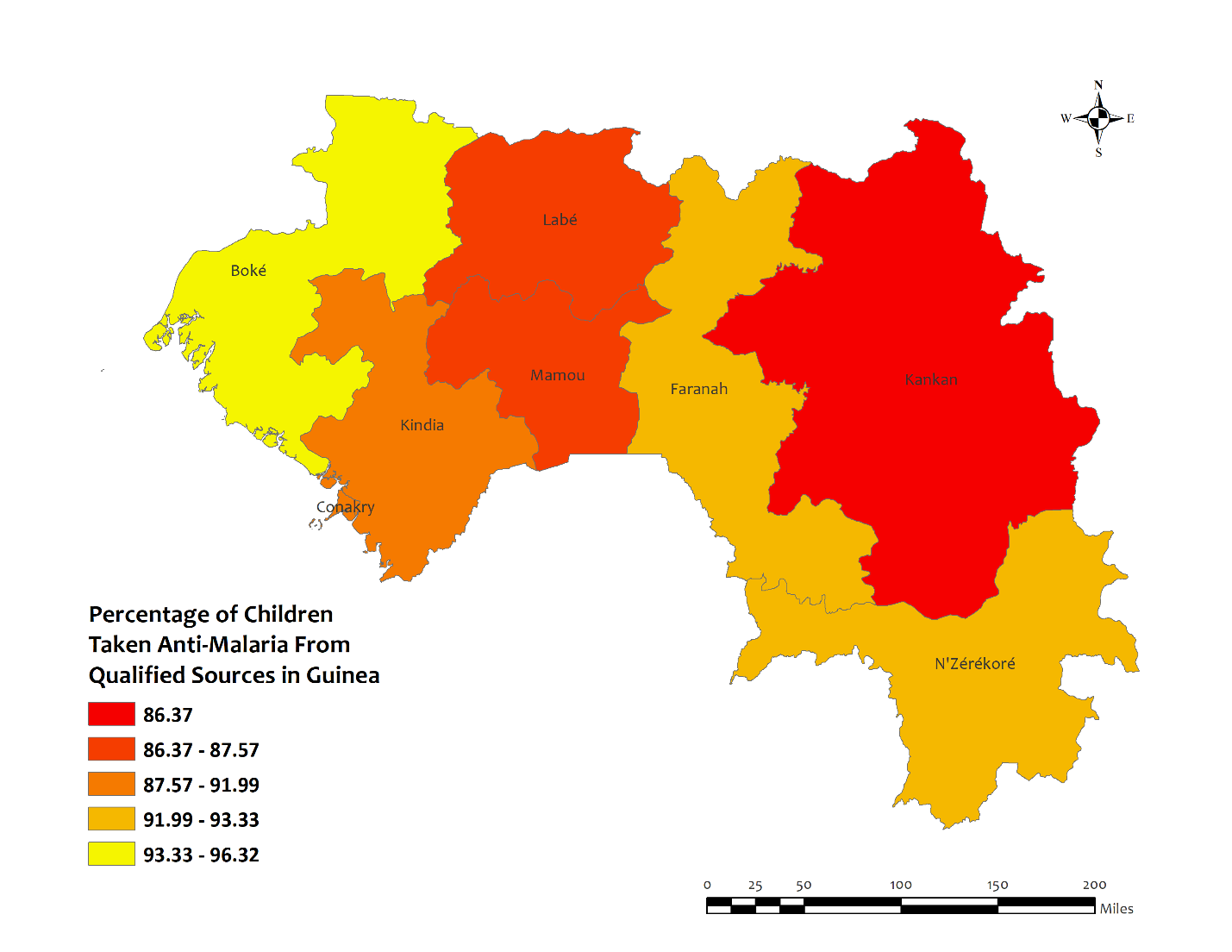


***Legend:*** ***Here, the darker shades of red indicate unqualified sources of antimalarial.*** ***Basemap data provided by ArcGIS (source:***[***https://hub.arcgis.com/datasets/esri::world-countries-generalized/explore?location=76.272878%2C-132.879863%2C12.88***](https://hub.arcgis.com/datasets/esri::world-countries-generalized/explore?location=76.272878%2C-132.879863%2C12.88)***)***

**Supplementary figure 3:** Region-wise Prevalence of Qualified Prescription of Antimalarial from Qualified Sources in Madagascar


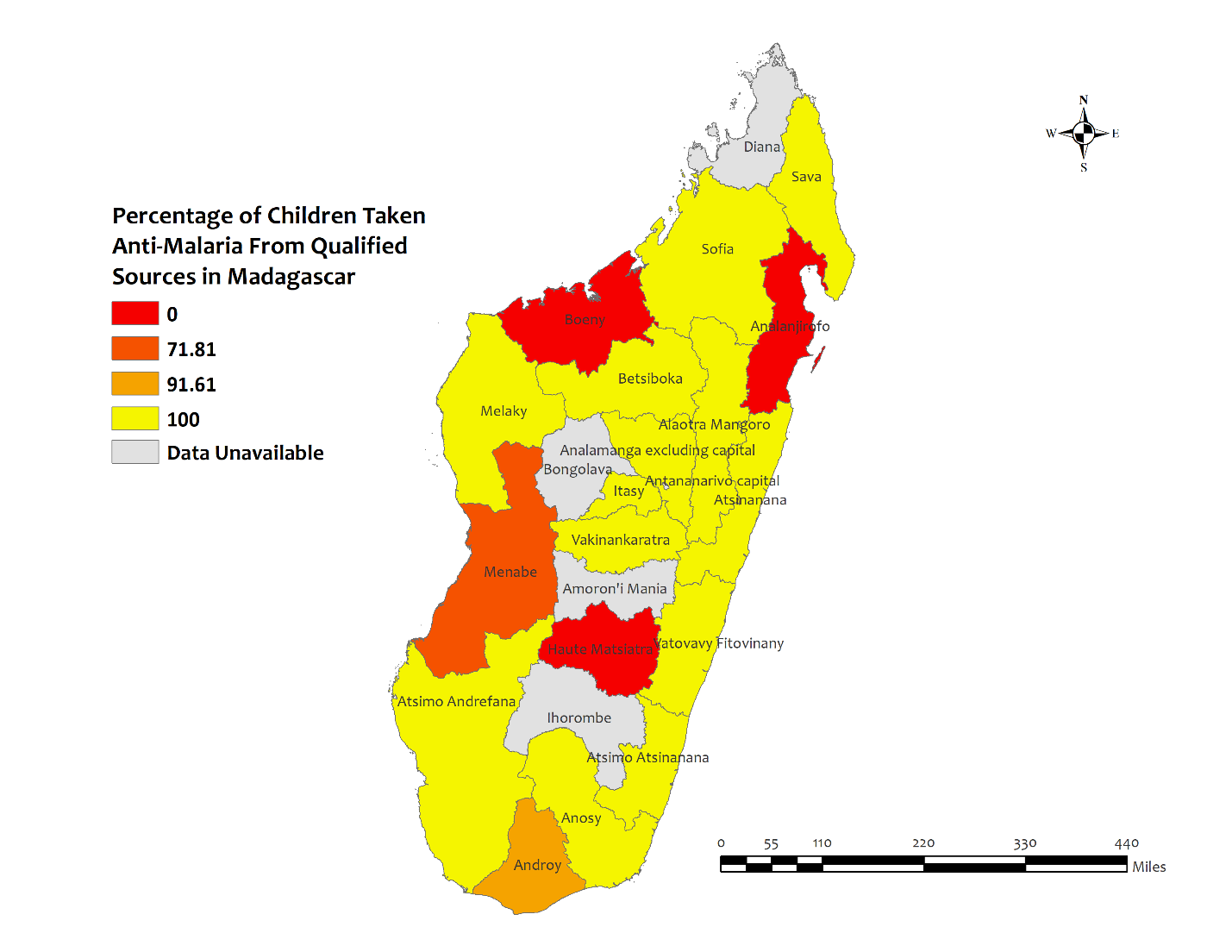


***Legend:*** ***Here, the darker shades of red indicate unqualified sources of antimalarial.*** ***Basemap data provided by ArcGIS (source:***[***https://hub.arcgis.com/datasets/esri::world-countries-generalized/explore?location=76.272878%2C-132.879863%2C12.88***](https://hub.arcgis.com/datasets/esri::world-countries-generalized/explore?location=76.272878%2C-132.879863%2C12.88)***)***

**Supplementary figure 4:** Region-wise Prevalence of Qualified Prescription of Antimalarial from Qualified Sources in Malawi


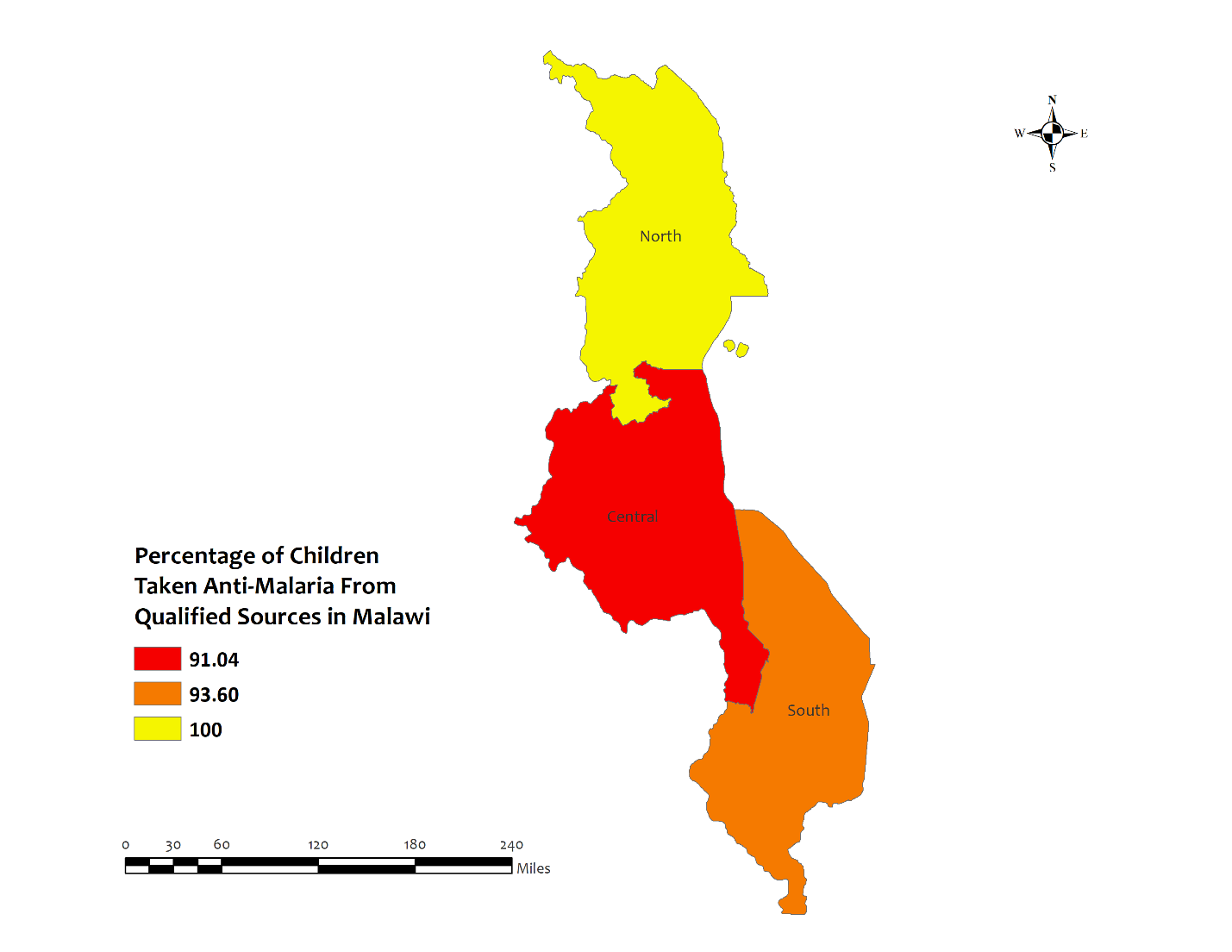


***Legend:*** ***Here, the darker shades of red indicate unqualified sources of antimalarial.*** ***Basemap data provided by ArcGIS (source:***[***https://hub.arcgis.com/datasets/esri::world-countries-generalized/explore?location=76.272878%2C-132.879863%2C12.88***](https://hub.arcgis.com/datasets/esri::world-countries-generalized/explore?location=76.272878%2C-132.879863%2C12.88)***)***

**Supplementary figure 5:** Region-wise Prevalence of Qualified Prescription of Antimalarial from Qualified Sources in Mali


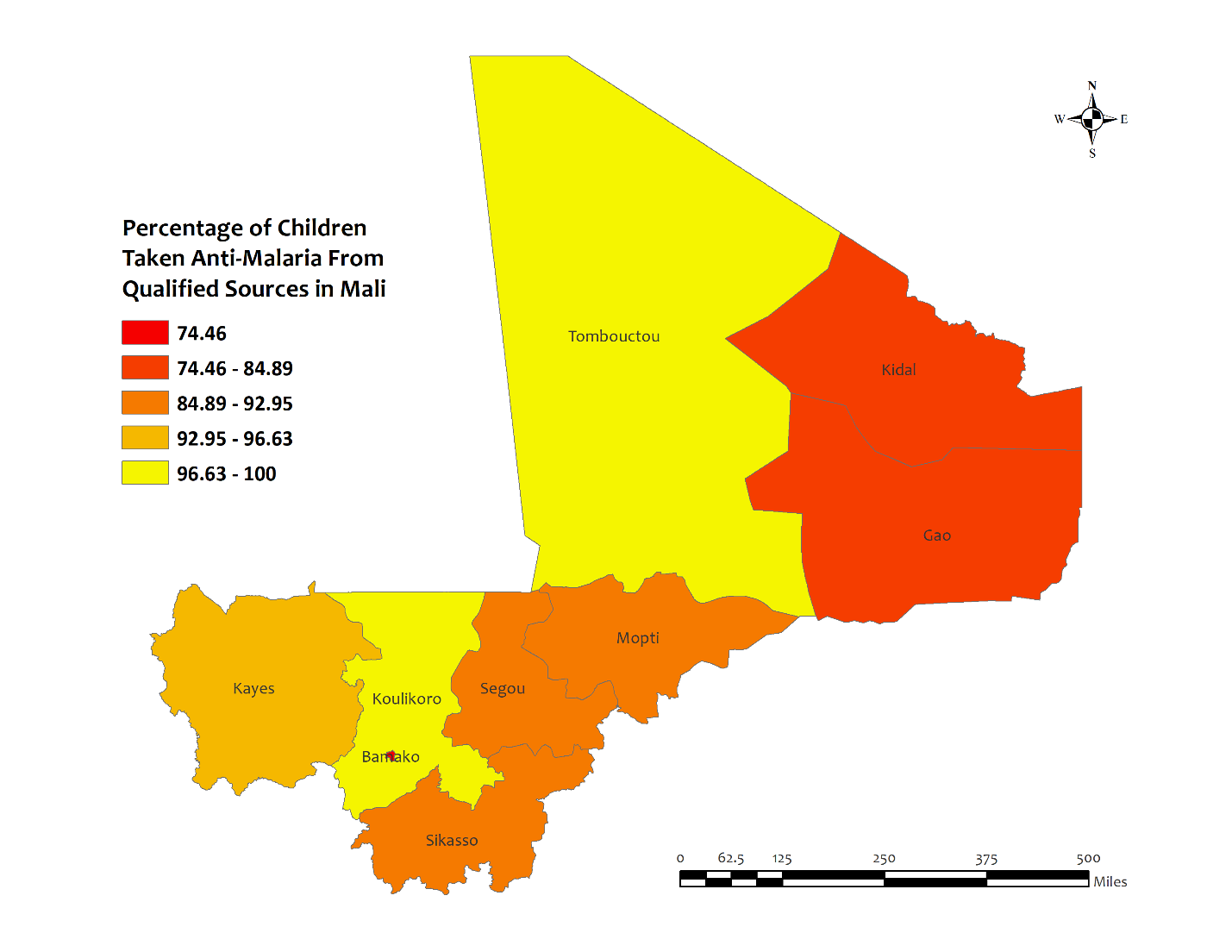


***Legend:*** ***Here, the darker shades of red indicate unqualified sources of antimalarial.*** ***Basemap data provided by ArcGIS (source:***[***https://hub.arcgis.com/datasets/esri::world-countries-generalized/explore?location=76.272878%2C-132.879863%2C12.88***](https://hub.arcgis.com/datasets/esri::world-countries-generalized/explore?location=76.272878%2C-132.879863%2C12.88)***)***

**Supplementary figure 6:** Region-wise Prevalence of Qualified Prescription of Antimalarial from Qualified Sources in Rwanda


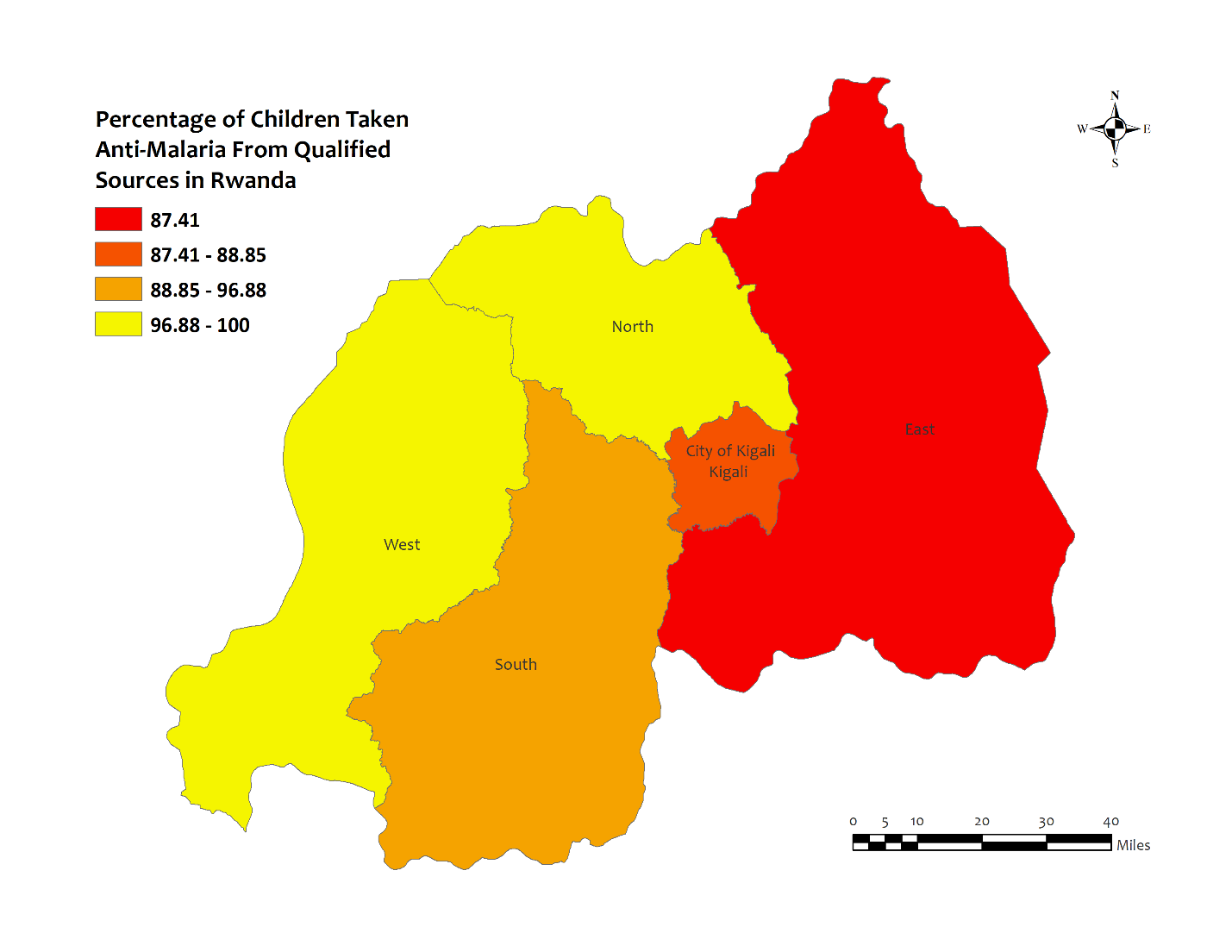


***Legend:*** ***Here, the darker shades of red indicate unqualified sources of antimalarial. Basemap data provided by ArcGIS (source:***[***https://hub.arcgis.com/datasets/esri::world-countries-generalized/explore?location=76.272878%2C-132.879863%2C12.88***](https://hub.arcgis.com/datasets/esri::world-countries-generalized/explore?location=76.272878%2C-132.879863%2C12.88)***)***

**Supplementary figure 7:** Region-wise Prevalence of Qualified Prescription of Antimalarial from Qualified Sources in Togo


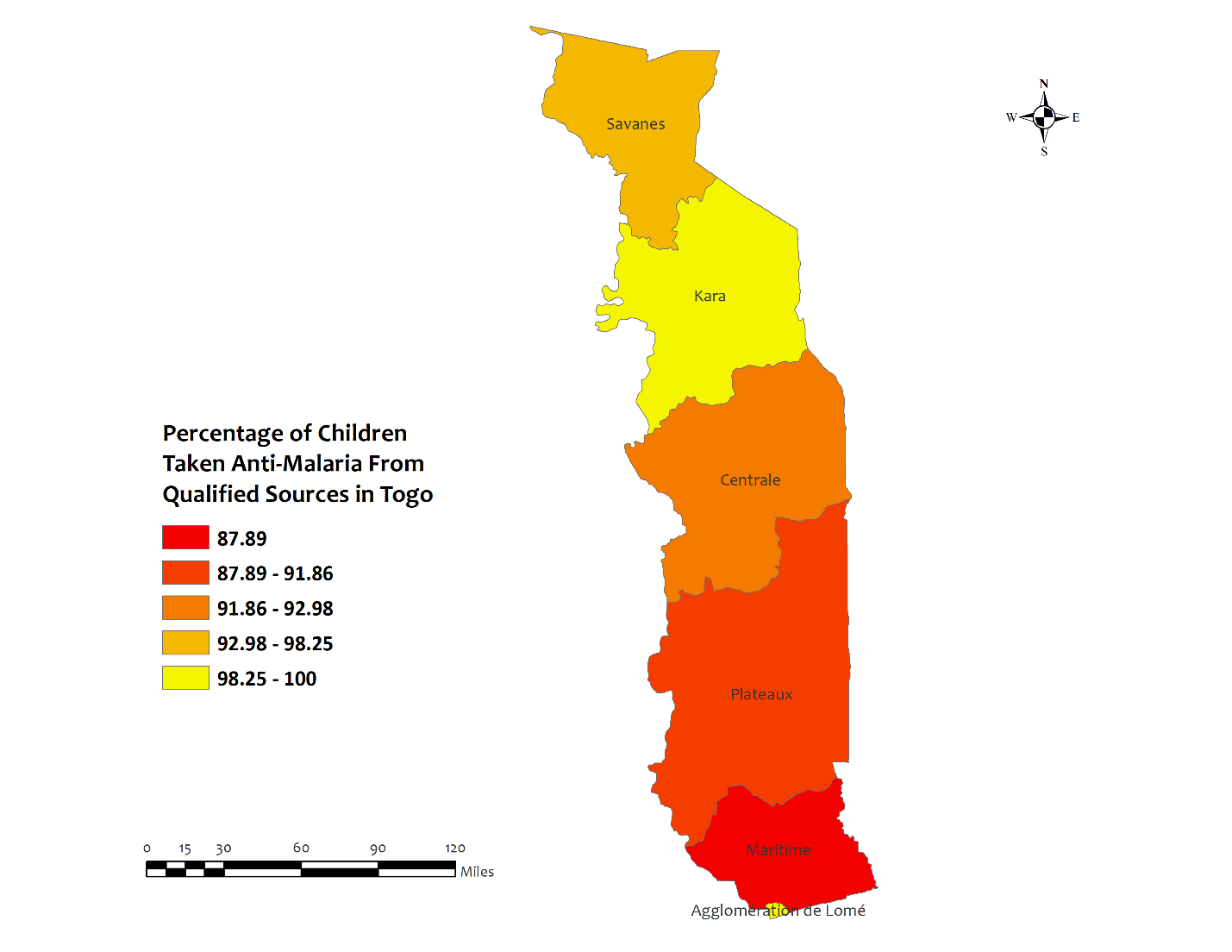


***Legend:*** ***Here, the darker shades of red indicate unqualified sources of antimalarial****.* ***Basemap data provided by ArcGIS (source:***[***https://hub.arcgis.com/datasets/esri::world-countries-generalized/explore?location=76.272878%2C-132.879863%2C12.88***](https://hub.arcgis.com/datasets/esri::world-countries-generalized/explore?location=76.272878%2C-132.879863%2C12.88)***)***

**Supplementary figure 8:** Region-wise Prevalence of Qualified Prescription of Antimalarial from Qualified Sources in Uganda


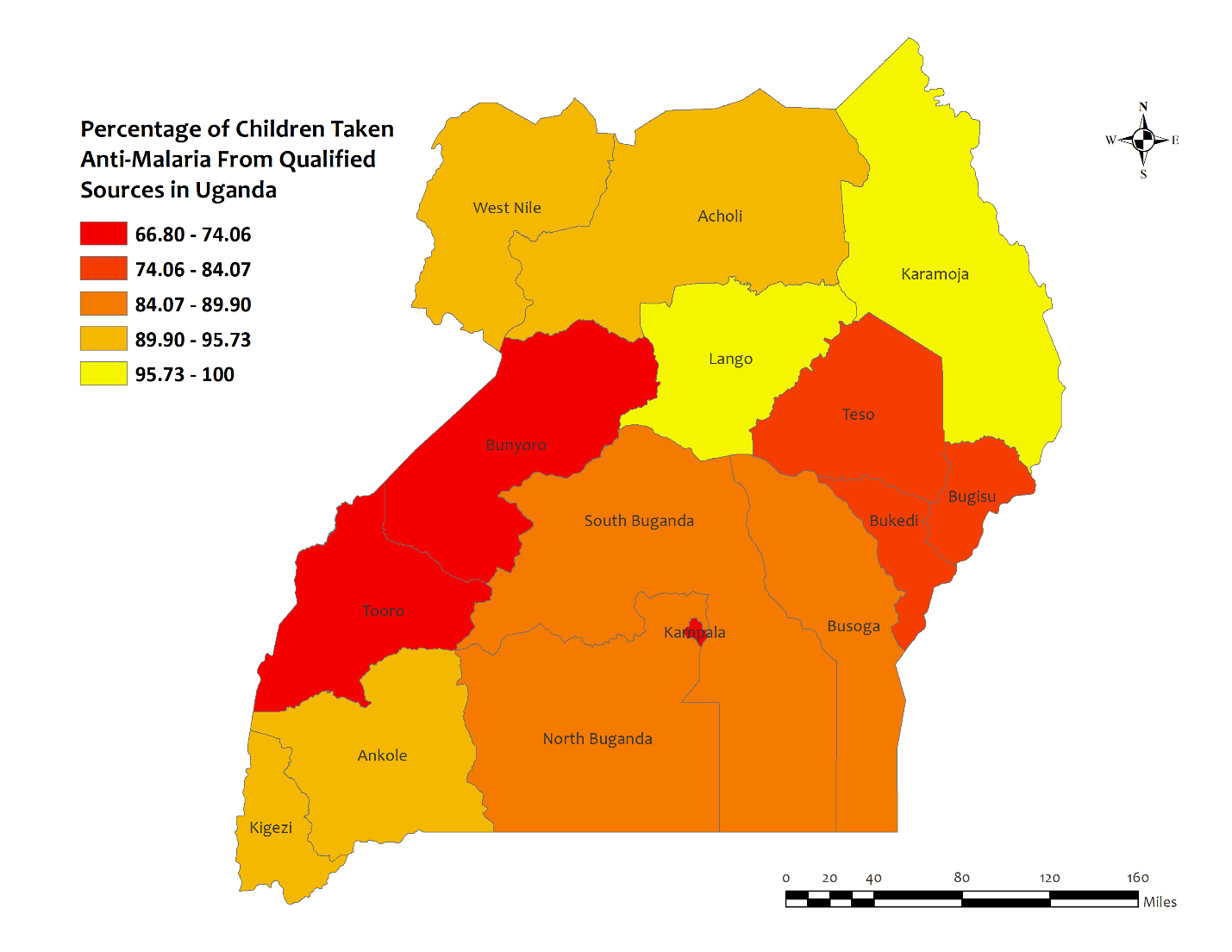


***Legend:*** ***Here, the darker shades of red indicate unqualified sources of antimalarial.*** ***Basemap data provided by ArcGIS (source:***[***https://hub.arcgis.com/datasets/esri::world-countries-generalized/explore?location=76.272878%2C-132.879863%2C12.88***](https://hub.arcgis.com/datasets/esri::world-countries-generalized/explore?location=76.272878%2C-132.879863%2C12.88)***)***

**Supplementary figure 9:** Region-wise Prevalence of Qualified Prescription of Antimalarial from Qualified Sources in Zambia


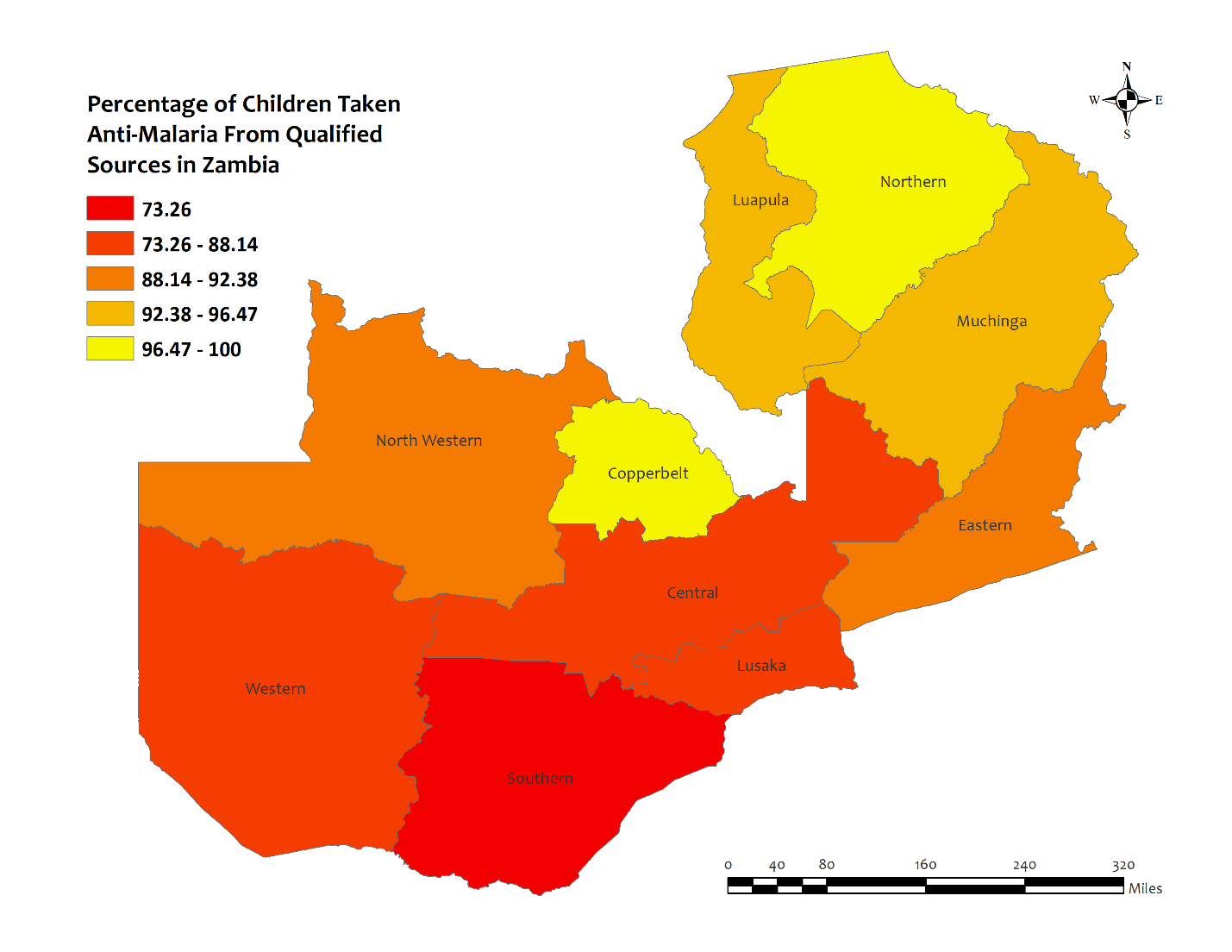


***Legend:*** ***Here, the darker shades of red indicate unqualified sources of antimalarial.*** ***Basemap data provided by ArcGIS (source:***[***https://hub.arcgis.com/datasets/esri::world-countries-generalized/explore?location=76.272878%2C-132.879863%2C12.88***](https://hub.arcgis.com/datasets/esri::world-countries-generalized/explore?location=76.272878%2C-132.879863%2C12.88)***)***
