# Supplementary material for: Prevalence of unqualified sources of antimalarial drug prescription for children under the age of five: A study in 19 low- and middle-income countries
Source: PLoS One. 2024 Mar 21;19(3):e0300347. doi: 10.1371/journal.pone.0300347 (PMC10956821; doi:10.1371/journal.pone.0300347)
Supplement: Attachment — Submitted filename: Response to Reviewers (1).docx [file pone.0300347.s004.docx]

## Review Responses

**ACADEMIC EDITOR**

1. As part of the manuscript submission process for PLOS ONE, we kindly request that you include the source of any basemaps and provide attribution to ArcGIS in the figure caption or within the manuscript. This is an important requirement as per the PLOS ONE copyright policy (https://journals.plos.org/plosone/s/licenses-and-copyright#loc-licenses-for-data).

For maps created using ArcGIS, please include a statement similar to the following (or whichever is applicable) in your figure caption or within the manuscript: “Basemap data provided by ArcGIS (source: URL if applicable).” If the map is a significant part of the research, consider including a more detailed description of the map source in the Methods section.

**Response:** *In alignment with PLOS ONE's copyright policy, we wish to confirm our adherence to the requirement for proper attribution of basemaps used in our figures. The basemap data employed in crafting our maps originates from ArcGIS, and for transparency and reference, we have included the specific source link. Within each figure legend, the following attribution has been incorporated: "Basemap data provided by ArcGIS (source:* [*https://hub.arcgis.com/datasets/esri::world-countries-generalized/explore?location=76.272878%2C-132.879863%2C12.88*](https://hub.arcgis.com/datasets/esri::world-countries-generalized/explore?location=76.272878%2C-132.879863%2C12.88)*)."*

1. We note that you have indicated that data from this study are available upon request. PLOS only allows data to be available upon request if there are legal or ethical restrictions on sharing data publicly. For more information on unacceptable data access restrictions, please see http://journals.plos.org/plosone/s/data-availability#loc-unacceptable-data-access-restrictions.

In your revised cover letter, please address the following prompts:

a) If there are ethical or legal restrictions on sharing a de-identified data set, please explain them in detail (e.g., data contain potentially sensitive information, data are owned by a third-party organization, etc.) and who has imposed them (e.g., an ethics committee). Please also provide contact information for a data access committee, ethics committee, or other institutional body to which data requests may be sent.

b) If there are no restrictions, please upload the minimal anonymized data set necessary to replicate your study findings as either Supporting Information files or to a stable, public repository and provide us with the relevant URLs, DOIs, or accession numbers. For a list of acceptable repositories, please see http://journals.plos.org/plosone/s/data-availability#loc-recommended-repositories.

We will update your Data Availability statement on your behalf to reflect the information you provide.

**Response:** *Regarding ethical or legal restrictions, we want to clarify that there are no such limitations on sharing the de-identified dataset associated with our study. The data we have generated do not contain sensitive information, and there are no third-party ownership issues or external restrictions imposed by ethics committees or other institutional bodies. As such, we are fully committed to making the dataset freely available for replication and further analysis. To fulfill the requirements, we have uploaded the minimal anonymized dataset necessary to replicate our study findings. The dataset is provided as Supporting Information files along with our revised submission.*

2. We note that Figures 2, 3, 4, 5, 6, 7, 8, 9, 10, 11, and 12 in your submission contain [map/satellite] images which may be copyrighted. All PLOS content is published under the Creative Commons Attribution License (CC BY 4.0), which means that the manuscript, images, and Supporting Information files will be freely available online, and any third party is permitted to access, download, copy, distribute, and use these materials in any way, even commercially, with proper attribution. For these reasons, we cannot publish previously copyrighted maps or satellite images created using proprietary data, such as Google software (Google Maps, Street View, and Earth). For more information, see our copyright guidelines: http://journals.plos.org/plosone/s/licenses-and-copyright.

We require you to either (1) present written permission from the copyright holder to publish these figures specifically under the CC BY 4.0 license, or (2) remove the figures from your submission:

1. You may seek permission from the original copyright holder of Figures 2, 3, 4, 5, 6, 7, 8, 9, 10, 11, and 12 to publish the content specifically under the CC BY 4.0 license.

We recommend that you contact the original copyright holder with the Content Permission Form (http://journals.plos.org/plosone/s/file?id=7c09/content-permission-form.pdf) and the following text:

“I request permission for the open-access journal PLOS ONE to publish XXX under the Creative Commons Attribution License (CCAL) CC BY 4.0 (http://creativecommons.org/licenses/by/4.0/). Please be aware that this license allows unrestricted use and distribution, even commercially, by third parties. Please reply and provide explicit written permission to publish XXX under a CC BY license and complete the attached form.”

Please upload the completed Content Permission Form or other proof of granted permissions as an "Other" file with your submission.

In the figure caption of the copyrighted figure, please include the following text: “Reprinted from [ref] under a CC BY license, with permission from [name of publisher], original copyright [original copyright year].”

2. If you are unable to obtain permission from the original copyright holder to publish these figures under the CC BY 4.0 license or if the copyright holder’s requirements are incompatible with the CC BY 4.0 license, please either i) remove the figure or ii) supply a replacement figure that complies with the CC BY 4.0 license. Please check copyright information on all replacement figures and update the figure caption with source information. If applicable, please specify in the figure caption text when a figure is similar but not identical to the original image and is therefore for illustrative purposes only.

The following resources for replacing copyrighted map figures may be helpful:

USGS National Map Viewer (public domain): http://viewer.nationalmap.gov/viewer/

The Gateway to Astronaut Photography of Earth (public domain): http://eol.jsc.nasa.gov/sseop/clickmap/

Maps at the CIA (public domain): https://www.cia.gov/library/publications/the-world-factbook/index.html and https://www.cia.gov/library/publications/cia-maps-publications/index.html

NASA Earth Observatory (public domain): http://earthobservatory.nasa.gov/

Landsat: http://landsat.visibleearth.nasa.gov/

USGS EROS (Earth Resources Observatory and Science (EROS) Center) (public domain): http://eros.usgs.gov/#

Natural Earth (public domain): <http://www.naturalearthdata.com/>

**Response:** *Upon careful consideration, we would like to clarify that the maps in question were not sourced from online platforms such as Google Maps, Street View, or Earth. Instead, we generated these figures using GIS (Geographic Information System) software, and we affirm that we are the rightful owners of the data and images presented in these figures. As the original creators of the maps used in our submission, we confirm that these materials are not subject to any external copyright restrictions. Therefore, we believe they inherently meet the criteria for publication under the Creative Commons Attribution License (CC BY 4.0).*

1. **Methods**

- Even though the MIS received ethical clearance in the respective countries. It would have been good for the researchers to seek for an ethical clearance from their own institution.
 ***Responses:*** *Our study relies on secondary data obtained from existing free sources (https://dhsprogram.com/data/), and our institution exclusively provides ethical clearance for studies involving primary data collection. Given that the Malaria Indicator Survey (MIS) data we utilized had already received ethical clearance within the respective countries, seeking additional ethical clearance from our institution for the secondary data was not applicable. It's important to note that ethical considerations for secondary data often involve adherence to privacy and confidentiality standards during data analysis and reporting rather than seeking a separate clearance. Moreover, the records in the data file were completely anonymous, and it is not possible to identify respondents' identities. We ensured that our study strictly adhered to ethical standards in handling and interpreting the existing data, prioritizing the privacy and confidentiality of individuals involved in the original data collection process.*

- The reasons given by the authors on the absence of information related to « non response » is too light. Because translation could be used to translate documents in English and access to key information.

***Responses:*** *Sorry for the inconvenience. We have revised the sentence accordingly at lines: 220-221 and Page: 9*

*“The reason was that the MIS survey reports were not available online for all the countries and among those available, not all reports include information on non-response rates.”*

2. **Results:** Suggest improving the countries map by comparing the malaria patterns and the antimalaria prescriptions maps by country

***Response:*** *We employ GIS software to generate clear and precise maps for all countries in our study.*

3. **Discussion:** This sentence is repeated twice in two successive paragraphs

« Regulatory bodies in the country lack the necessary power to effectively control drug pricing or

curb the excessive usage of generic medicines. As a result, the availability of generic drugs is

limited, leading to a significant markup in prices. »

***Responses:*** *We have revised this section accordingly.*

**Review Comments to the Author**

Reviewer #1:

General comments
The authors should edit number in table 1 and Figure 1 and rework the discussion to focus it according to the goal of this study. The authors also should make more effort to summarize the sections to reduce the number of pages.
I appreciated this article because of its originality and recommended its publication provided the authors will do the revision according to our comments.

***Response:*** *We express our gratitude to the reviewers for their invaluable feedback aimed at enhancing the quality of our research. In response to their comments, we have made the necessary updates to Table 1 and Figure 1. Additionally, as per their suggestions, the discussion section has undergone a thorough revision to provide a more concise and summarized overview.*
**Abstract**
Background
Antimalarial resistance poses a severe danger to global health. add drug resistance.

***Response:*** *We have revised the abstract accordingly on lines: 27 and page: 2.*

*“Antimalarial drug resistance poses a severe danger to global health.”*
Findings
About 60% instead 40%

***Response:*** *We have revised the abstract accordingly on lines: 42 and page: 2.*

*“In several LMICs (Tanzania, Nigeria, and Ghana), a substantial portion of recent antimalarial prescriptions for malaria was taken from unqualified sources (about 60%).”*
**Introduction**
Introduction must be more focus with the tittle:
First describe malaria situation around the world emphasizing the part of children under five on malaria cases and death, the strategies of malaria diagnostic and treatment, the availability of the diagnostic tools and antimalarial drug and the drug resistance management plan.
The second section of the introduction must talk about the justification that unqualified treatments is significantly identified and will need to better documented to understand the causes and the possible effects including the threat in malaria drug resistance.

***Response:*** *We have revised the introduction following the suggestions provided by the reviewer on lines: 57-141 and pages 3-5.*
**Methods**
Methods have been well described according to data sources, sampling methods and data analyses.
Some inputs to take into account related to the narrative:
Data Gap Analysis on Antimalarial Prescriptions. In the future, surveys will be able to follow kids both throughout the cascade and after their treatment is finished to assess the outcomes of the causality of the child's quality of malaria care. This sentence must be moved to the Conclusion section.

***Response:*** *Thank you for your comments. We moved the sentence “In the future, surveys will be able to follow kids both throughout the cascade and after their treatment is finished to assess the outcomes of the causality of the child's quality of malaria care.” to the conclusion section.*

**Results
Descriptives Analysis**
Decimals must be broken into whole numbers in figure 1 and in the narrative.
Total in table 1 for each column doesn’t match with what we have in the text and in figure 1.
Had Fever Recently Antimalarial taken for Malaria Antimalarial taken from Qualified Sources,
Total 29480 11171 7522
In the narrative 29 478 9477 7511

Males make up 54035.8 of them, or 50.9%, while females make up 5229.2, or 48.1%. Not found nor in table 1 neither in Figure 1

***Response:*** *We updated the text and Figure 1 accordingly on lines:266-272 and pages:10.*

**Country Descriptives**
Page 17. In this section, we presented the region-wise prevalence of five countries with the highest antimalarial prescriptions from qualified sources for recent malarial fever in children under five. This section must be more summarized as it is expected that there would not be a big difference between zones.

***Response:*** *We have summarized this section on lines: 293-233 and pages:10-13.*

**Discussion**
P 14. The reasons for the high percentages of qualified prescriptions of antimalarial for recent malarial fever in children under five across the LMICs are manifold…….. P 22. Several studies highlight the shortcomings of ostensibly straightforward approaches (such as fever charts or RDTs) to properly manage fevers and drug usage at the community level45,46,47 . To be deleted and focus must in Africa, about malaria prescription and malaria drug consumption in children under five.

Below this section, from ‘’Moreover, several studies that found various factors that influence high rates of qualified prescriptions are insufficient diagnostic services, a lack of antibiotic guidelines, difficulty in monitoring patient progress, unsatisfactory intensive care facilities in rural areas, patient demand for immediate relief, apparent patient anticipations from prior prescriptions, using up production, and apprehension about losing patients to competition’’, this is appreciated to be in the discussion.

**Response:** *We removed section “P14. The reasons for the high percentages of qualified prescriptions of antimalarial for recent malarial fever in children under five across the LMICs are manifold…….. P 22.”and keep from ‘’Moreover, several studies that found various factors that influence high rates of qualified prescriptions are insufficient diagnostic services, a lack of antibiotic guidelines, difficulty in monitoring patient progress, unsatisfactory intensive care facilities in rural areas, patient demand for immediate relief, apparent patient anticipations from prior prescriptions, using up production, and apprehension about losing patients to competition’’ on lines*

The discussion must be reworked to follow the different points:
• Had Fever Recently,
• Antimalarial taken for Malaria
• Antimalarial taken from Qualified Sources
I putsome aspects that would be taken into account in the discussion et recommendations.
Antimalarial taken for Malaria
**Discussion**
For the high percentages, it will be necessary to ascertain whether they are suspected or confirmed cases.
If su*spected, are there national* guidelines for the diagnosis and treatment of malaria?
If so, why are they not respected?
If not, what are the constraints for having the guidelines?
If malaria is confirmed, does the low-test positivity rate justify the low percentages of antimalarial drug use?

***Response:*** *We have added a section in the discussion as suggested by the reviewers on lines:515-524 and pages 19-20.*

*“The high percentages of antimalarial prescriptions for recent malarial fever in children under five are suspected cases. It is essential to explore the existence and adherence to national guidelines for the diagnosis and treatment of malaria. Several studies have highlighted challenges in implementing guidelines, including ineffective testing, inefficient malaria care, and the administration of antimalarials without proper blood tests, potentially contributing to overprescription^28-29^. A study in Sub-Saharan Africa found that overprescription was linked to limited diagnostic competence, reliance on clinical symptoms, and noncompliance with treatment recommendations^34^. Addressing these issues is crucial to ensuring that antimalarials are prescribed judiciously, avoiding unnecessary use and potential development of resistance ^18,46-47^.”*

**Recommendations**
Make available guidelines for the management of malaria cases and their application
Require confirmation of malaria cases before prescribing antimalarials
Antimalarial taken from Qualified Sources
Discussion
For the low, it will be necessary to try to find the main causes for each country concerned.
Assessment of the level of knowledge of malaria by the population and the control interventions?
Are there communication strategies for early use of health facilities?
Are inputs for malaria diagnosis and treatment available and accessible?
Level of collaboration between traditional practitioners and the Ministry of Health?
Recommendations
Strengthen communication regarding the use of the nearest health facilities. Implement sustainable strategies capable of counteracting non-compliant treatment causes.
Make available and permanent diagnostic inputs and medicines for the treatment of malaria cases.
Making health care delivery *points* accessible.

***Response:*** *We adjusted the recommendation section based on the reviewer's feedback on lines:526-554 and pages: 20-21*

*“In Sub-Saharan African countries, it is imperative to implement a comprehensive approach for the effective management of malaria cases. Clear and updated guidelines should be developed and disseminated widely, emphasizing the importance of confirming malaria cases before prescribing antimalarials, with healthcare professionals receiving adequate training. Rigorous quality assurance measures must be in place to ensure that antimalarial medications are sourced exclusively from qualified and reputable suppliers. Understanding the root causes of malaria prevalence in each country is crucial, requiring comprehensive studies that account for regional variations. Regular assessments should be conducted to gauge the population's knowledge of malaria and the effectiveness of existing control interventions, with tailored health education programs addressing awareness gaps. Communication strategies promoting early utilization of health facilities, the availability of diagnostic tools, and collaboration with traditional practitioners are vital components. Sustainable strategies to counteract non-compliance with treatment regimens and efforts to improve healthcare delivery point accessibility, particularly in rural areas, are essential. Strengthening communication about the nearest health facilities and ensuring a reliable supply chain for diagnostic inputs and medicines are paramount for successful malaria management in the region.”*
