# Supplementary material for: Prevalence of unqualified sources of antimalarial drug prescription for children under the age of five: A study in 19 low- and middle-income countries
Source: PLoS One. 2024 Mar 21;19(3):e0300347. doi: 10.1371/journal.pone.0300347 (PMC10956821; doi:10.1371/journal.pone.0300347)
Supplement: Attachment — Submitted filename: Reviewer DiopA.docx [file pone.0300347.s005.docx]

**Prevalence of unqualified sources of antimalarial drug prescription for children under the age of five: a study in 19 low- and middle-income countries**

**Reviewer: Abdoulaye Diop**

**FIRST REVIEW**

Please find below my comments

**General comments**

The authors should edit number in table 1 and Figure 1 and rework the discussion to focus it according the goal of this study. The authors also should make more effort to summarize the sections to reduce the number of pages.

I appreciated this article because of its originality and recommended its publication provided the authors will do the revision according our comments.

**Abstract**

Background

*Antimalarial resistance poses a severe danger to global health.* **add drug resistance**

Findings

About 60% instead 40%

**Introduction**

**Introduction must be more focus with the tittle:**

First describe malaria situation around the world emphasizing the part of children under five on malaria cases and death, the strategies of malaria diagnostic and treatment, the availability of the diagnostic tools and antimalarial drug and the drug resistance management plan.

The second section of the introduction must talk about the justification that unqualified treatments is significantly identified and will need to better documented to understand the causes and the possible effects including the threat in malaria drug resistance.

**Methods**

Methods have been well described according data sources, sampling methods and data analyses.

Some inputs to take into account related to the narrative:

Data Gap Analysis on Antimalarial Prescriptions. *In the future, surveys will be able to follow kids both throughout the cascade and after their treatment is finished to assess the outcomes of the causality of the child's quality of malaria care.* This sentence must be moved to the Conclusion section

**Results**

**Descriptives Analysis**

Decimals must be broken into whole numbers in figure 1 and in the narrative.

Total in table 1 for each column doesn’t match with what we have in the text and in figure 1.

| **Country** | **Had Fever Recently, Weighted N (%)** | **Antimalarial taken for Malaria, Weighted N (%)** | **Antimalarial taken from Qualified Sources, Weighted N (%)** |
| --- | --- | --- | --- |
| Angola | 2632 | 706 | 656 |
| Burkina Faso | 1176 | 600 | 557 |
| Burundi | 1806 | 455 | 404 |
| Ghana | 812 | 370 | 198 |
| Guinea | 910 | 290 | 236 |
| Kenya | 559 | 15 | 12 |
| Liberia | 1011 | 642 | 446 |
| Madagascar | 1087 | 68 | 51 |
| Malawi | 1038 | 305 | 248 |
| Mali | 2494 | 780 | 684 |
| Mozambique | 1458 | 1458 | 422 |
| Nigeria | 3917 | 800 | 395 |
| Rwanda | 884 | 884 | 153 |
| Senegal | 1654 | 44 | 44 |
| Sierra Leone | 1535 | 868 | 764 |
| Tanzania | 1429 | 505 | 254 |
| Togo | 748 | 225 | 190 |
| Uganda | 1715 | 1074 | 883 |
| Zambia | 2616 | 1081 | 924 |
| **Total** | **29480** | **11171** | **7522** |
| **In the narrative** | **29 478** | **9477** | **7511** |

*Males make up 54035.8 of them, or 50.9%, while females make up 5229.2, or 48.1%*. Not found nor in table 1 neither in Figure 1

**Country Descriptives**

Page 17. *In this section, we presented the region-wise prevalence of five countries with the highest antimalarial prescriptions from qualified sources for recent malarial fever in children under five*. This section must be more summarized as it is expected that there would not be a big difference between zones.

**Discussion**

*P 14. The reasons for the high percentages of qualified prescriptions of antimalarial for recent malarial fever in children under five across the LMICs are manifold…….. P 22. Several studies highlight the shortcomings of ostensibly straightforward approaches (such as fever charts or RDTs) to properly manage fevers and drug usage at the community level45,46,47 .* To be deleted and focus must in Africa, about malaria prescription and malaria drug consumption in children under five.

Below this section, from ‘’Moreover, several studies that found various factors that influence high rates of qualified prescriptions are insufficient diagnostic services, a lack of antibiotic guidelines, difficulty in monitoring patient progress, unsatisfactory intensive care facilities in rural areas, patient demand for immediate relief, apparent patient anticipations from prior prescriptions, using up production, and apprehension about losing patients to competition’’, this is appreciated to be in the discussion.

The discussion must be reworked to follow the different points:

- Had Fever Recently,
- Antimalarial taken for Malaria
- Antimalarial taken from Qualified Sources

I put in this table below some aspects that would be taken into account in the discussion et recommendations.

| Country | Had Fever Recently, Weighted N (%) | Antimalarial taken for Malaria, Weighted N (%) | Antimalarial taken from Qualified Sources, Weighted N (%) |
| --- | --- | --- | --- |
| Angola | 2632 (34.4) | 706.1 (95.0) | 656.3 (98.7) |
| Burkina Faso | 1176 (20.3) | 600.2 (99.7) | 556.8 (97.4) |
| Burundi | 1806 (35.5) | 455.4 (98.2 | 404.4 (92.1) |
| Ghana | 811.5 (30.1) | 370.2 (99.8) | 197.8 (61.1) |
| Guinea | 909.9 (23.3) | 289.7 (31.8) | 235.8 (91.5) |
| Kenya | 559.2 (17.4) | 14.78 (2.6) | 11.51 (83.4) |
| Liberia | 1011 (40) | 642.2 (97.0) | 446.4 (75.0) |
| Madagascar | 1087 (16.1) | 68.33 (61.7) | 51.36 (87.8) |
| Malawi | 1038 (40.7) | 305.4 (99.8) | 247.7 (92.4) |
| Mali | 2494 (27.3) | 779.7 (31.3) | 684.2 (93.3) |
| Mozambique | 1458 (31.3) | 1458 (100) | 422.3 (98.2) |
| Nigeria | 3917 (36.8) | 799.5 (20.4) | 394.8 (60.0) |
| Rwanda | 884.1 (31.1) | 884.1 (100) | 153.3 (92.0) |
| Senegal | 1654 (30.0) | 44.0(2.7) | 44 (100) |
| Sierra Leone | 1535 (27.1) | 868.2 (99.4) | 763.9 (96.6) |
| Tanzania | 1429 (20.6) | 504.8 (99.3) | 254 (59.9) |
| Togo | 747.5 (24.4) | 225.2(98.4) | 190.1 (94.6) |
| Uganda | 1715 (27.1) | 1074 (98.8) | 883.4 (87.8) |
| Zambia | 2616 (21.4) | 1081 (99.5) | 924.4 (93.2 |
| **Discussion** |  | For the high percentages, it will be necessary to ascertain whether they are suspected or confirmed cases.  If suspected, are there national guidelines for the diagnosis and treatment of malaria?  If so, why are they not respected?  If not, what are the constraints for having the guidelines?  If malaria is confirmed, does the low-test positivity rate justify the low percentages of antimalarial drug use? | For the low, it will be necessary to try to find the main causes for each country concerned.  Assessment of the level of knowledge of malaria by the population and the control interventions?  Are there communication strategies for early use of health facilities?  Are inputs for malaria diagnosis and treatment available and accessible?  Level of collaboration between traditional practitioners and the Ministry of Health? |
| **Recommendations** |  | Make available guidelines for the management of malaria cases and their application  Require confirmation of malaria cases before prescribing antimalarials | Strengthen communication regarding the use of the nearest health facilities. Implement sustainable strategies capable of counteracting non-compliant treatment causes.  Make available and permanent diagnostic inputs and medicines for the treatment of malaria cases.  Making health care delivery points accessible |

Antimalarial taken for Malaria

Discussion

For the high percentages, it will be necessary to ascertain whether they are suspected or confirmed cases.

If suspected, are there national guidelines for the diagnosis and treatment of malaria?

If so, why are they not respected?

If not, what are the constraints for having the guidelines?

If malaria is confirmed, does the low-test positivity rate justify the low percentages of antimalarial drug use?

Recommendations

Make available guidelines for the management of malaria cases and their application

Require confirmation of malaria cases before prescribing antimalarials

Antimalarial taken from Qualified Sources

Discussion

For the low, it will be necessary to try to find the main causes for each country concerned.

Assessment of the level of knowledge of malaria by the population and the control interventions?

Are there communication strategies for early use of health facilities?

Are inputs for malaria diagnosis and treatment available and accessible?

Level of collaboration between traditional practitioners and the Ministry of Health?

Recommendations

Strengthen communication regarding the use of the nearest health facilities. Implement sustainable strategies capable of counteracting non-compliant treatment causes.

Make available and permanent diagnostic inputs and medicines for the treatment of malaria cases.

Making health care delivery points accessible

**SECOND REVIEW**

Move Fig 13 to fig 2. Also add the number of male and female in the figure.
